# Antimicrobial Activity of Chitosan-Carbon Nanotube Hydrogels

**DOI:** 10.3390/ma7053946

**Published:** 2014-05-19

**Authors:** Jayachandran Venkatesan, Rangasamy Jayakumar, Annapoorna Mohandas, Ira Bhatnagar, Se-Kwon Kim

**Affiliations:** 1 Marine Bioprocess Research Center and Department of Marine-Bio. Convergence Science, Pukyong National University, Busan 608-737, Korea; E-Mail: venkatjchem@pknu.ac.kr; 2 Amrita Center for Nanosciences and Molecular Medicine, Amrita Institute of Medical Sciences and Research Centre, Kochi682041, India; E-Mails: rjayakumar@aims.amrita.edu (R.J.); annapoornamohandas@aims.amrita.edu (A.M.); 3 Nanotheranostics Laboratory, Centre for Cellular and Molecular Biology, Hyderabad 500-007, India; E-Mail: ibhatnagar@gmail.com

**Keywords:** marine biomaterials, tissue engineering, chitosan, carbon nanotube, antimicrobial activity

## Abstract

In the present study, we have prepared chitosan-carbon nanotube (Chitosan-CNT) hydrogels by the freeze-lyophilization method and examined their antimicrobial activity. Different concentrations of CNT were used in the preparation of Chitosan-CNT hydrogels. These differently concentrated CNT hydrogels were chemically characterized using Fourier Transform-Infrared Spectroscopy, Scanning Electron Microscopy and Optical microscopy. The porosity of the hydrogels were found to be >94%. Dispersion of chitosan was observed in the CNT matrix by normal photography and optical microscopy. The addition of CNT in the composite scaffold significantly reduced the water uptake ability. In order to evaluate antimicrobial activity, the serial dilution method was used towards *Staphylococcus aureus*, *Escherichia coli* and *Candida tropicalis*. The composite Chitosan-CNT hydrogel showed greater antimicrobial activity with increasing CNT concentration, suggesting that Chitosan-CNT hydrogel scaffold will be a promising biomaterial in biomedical applications.

## 1. Introduction

Microbial infections cause serious risk to human life with chronic obstructive pulmonary disease, which is the fourth leading cause of death in the United States [1]. Nanoparticles such as silver/copper [2] and carbon nanotube (CNT) [3] play a vital role in the control of microbial growth. Among all the nanoparticles, CNT has unique features such as optical, electronic, magnetic, chemical and mechanical properties, which enable us to use CNT in various applications including wound dressing, tissue engineering, biosensing and drug delivery. Low metal content CNT has been proven to exhibit strong antimicrobial activity [3]. Chemical functionalization of CNT brings new properties, increases the antimicrobial activity and decreases the toxicity when checked on mammalian cells. CNT/poly(l-lysine)/poly(l-glutamic acid) thin film exhibits significant antimicrobial activity (up to 90%) towards *E. coli* and *Staphyloccoccus epidermidis* [4,5]. Covalent attachment of epilson-polylysine with CNT exhibited enhanced antimicrobial activities against *E. coli*, *Pseudomonas aeruginosa* and *S. aureus*. Silver-CNT composite showed strong antibacterial activity against *E. coli* in the dark [6]. Since the dispersion of CNT is problematic, the usage of CNT in the microbial treatment is limited. In addition, the direct contact of CNT aggregates leads to cell damage, consequently causing cell death [7]. Chemical purification or functionalization of CNT can alter the physiochemical properties which makes it dispersive or easy to dissolve in various solvents [8].

Chitosan is a linear polysaccharide, consisting of d-glucosamine and *N*-acetyl glucosamine unit, which is commonly isolated from marine waste of crustacean shells and shrimps [9]. Chitosan possesses several applications that are not only limited to biological and biomedical fields, for example: wound dressing, photography, cosmetics, artificial skin, food, nutrition, ophthalmology, water engineering, paper finishing, solid state batteries, drug delivery systems, tissue engineering, antibacterial activity, blood anticoagulant and fat trapper due to its biocompatibility and biodegradability [10]. Antibacterial activities of chitosan have been explained in previous reports, where various Gram negative and Gram positive bacteria have been used [11]. The mechanism of chitosan antibacterial activity is: positive charged chitosan moiety can interact with negatively charged bacterial cell surface, which causes Cell membrane interruption leading to death [11,12].

Recently, hydrogel based biomaterials have gained much attention in the research arena due to their physiological behavior similar to human tissue function. Among various hydrogels, chitosan-based hydrogel systems have been checked for antimicrobial activity [13,14]. Chitosan with oxalyl bis 4-(2,5-dioxo-2H-pyrrol-1(5H)-yl)benzamide hydrogels were prepared and studied for their antimicrobial activity against *Aspergillus fumigatus*, *A. niger*, *Bacillis subtilis*, *S. aureus*, *Streptococcus*, *Salmonella typhimurium* and *E. coli.* Chemically modified chitosan showed stronger antimicrobial activity when compared with crude chitosan [14]. In another study, chitosan, *N*,*O*-carboxymethylated chitosan and *O*-carboxymethylated chitosan showed significant antimicrobial activities towards *E. coli* [15]. Chitosan coated Ag-loaded nanosilica showed antibacterial activity against *E. coli* and *S. aureus* [16–18]. Another study reported that Chitosan based nanocomposite films showed significant antimicrobial activity [19]. Apart from this, in recent decades, carbon nanotube/chitosan biocomposites have been widely used in the medical field (tissue engineering, biosensors and drug delivery) [20,21].

In this study, we prepared a series of chitosan/carbon nanotube hydrogels and checked them for antimicrobial activity against *S. aureus*, *E. coli* and *Candida tropicalis*. Our main goal of this work is to combine the antimicrobial activity of chitosan and CNT in order to design biocompatible antimicrobial biomaterials.

## 2. Results and Discussion

A small amount of CNT in the polymer matrix makes great alterations in mechanical, electrical and thermal properties. However, the problem of the practical application of CNT is dispersion in water, organic solvents and polymer matrix. Generally, the dispersion of CNT can be improved by some of the pretreatment methods such as functionalization with strong acid (nitric acid and sulfuric acid mixture). We have used three different concentrations of CNT *viz*., 25, 50 and 100 mg. In our case, it was observed that the dispersion of CNT in chitosan hydrogel was uniform. This might be due to pi bond interaction of CNT with chitosan amino and hydroxyl groups. It was observed that by increasing the CNT concentration in the chitosan hydrogel, the black color intensifies. Under acidic condition (pH < 6), chitosan is soluble in water and acts as a kind of polyelectrolyte. This property makes chitosan a potential dispersing agent for CNT. Chitosan salt treated CNT were dispersed in different organic solvents such as hexane, isopropanol, toluene, dichloromethane, chloroform, tetrahydrofuran, ethanol, ethyl acetate and dimethyl sulfoxide [22].

### 2.1. Water Uptake and Retention Ability

**Figure 1.**
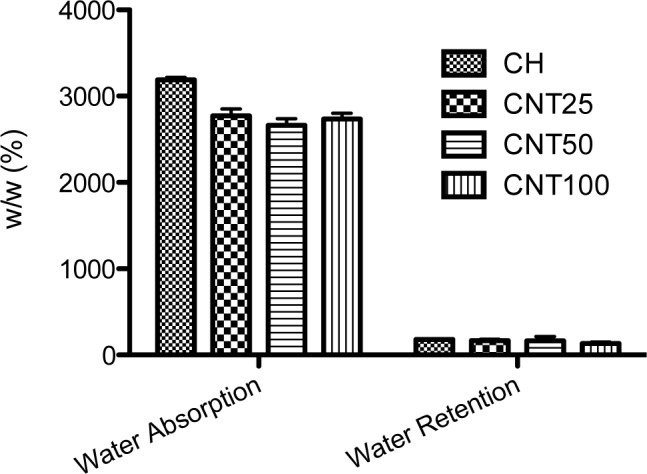
Water uptake and retention ability of chitosan, CNT25, CNT50 and CNT100 composite hydrogels after 24 h. The values are mean ± SD.

Chitosan is commonly soluble in acetic acid; the swelling behavior of prepared hydrogels was checked with PBS solution (Figure 1). From the data, the hydrogels swelled immediately because of the hydrophilic character of chitosan. The highest degree of swelling behavior was observed in raw chitosan and no difference has been observed between the CNT composite scaffolds. In addition, water uptake ability of CNT composite shows lower ability when compared to chitosan scaffold; this may be due to the hydrophobic nature of CNT. Further, there is no difference observed in water retention ability among the scaffolds.

### 2.2. Fourier Transform Infrared Spectroscopy

**Figure 2.**
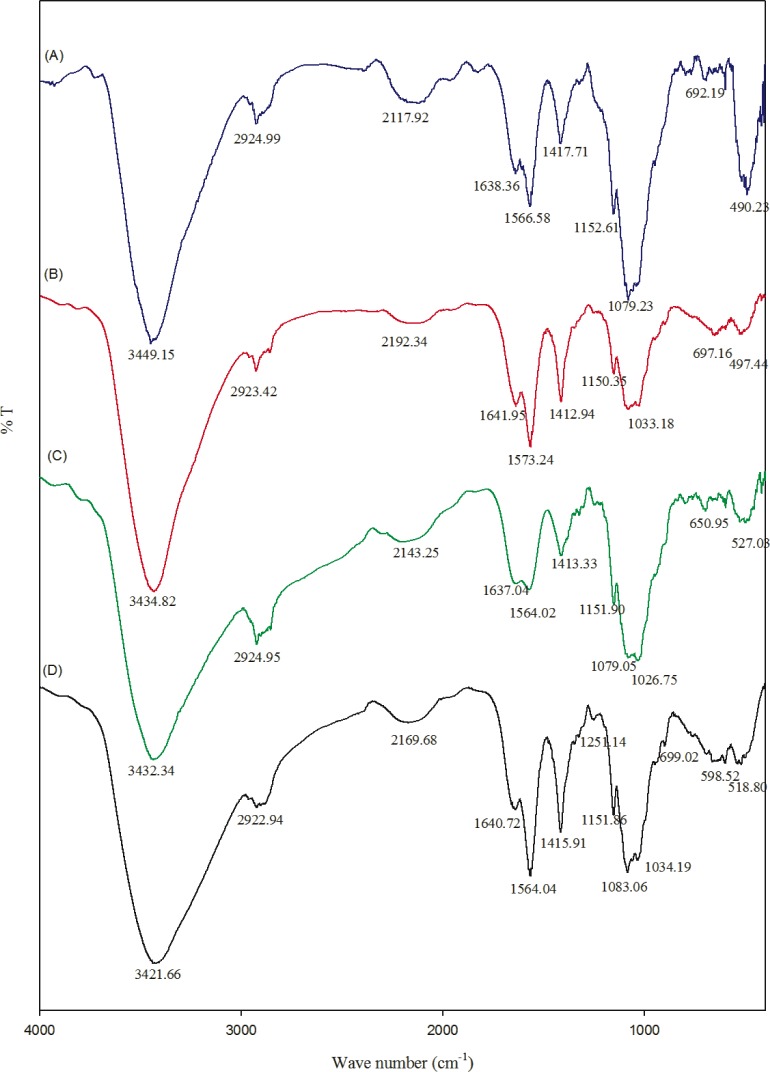
FT-IR spectrum of (**A**) chitosan; (**B**) CNT25; (**C**) CNT50; and (**D**) CNT100.

FT-IR spectra of chitosan and their composites are shown in the Figure 2. The FT-IR spectrum of chitosan showed the important peaks at 1151, 1083, 1034 and 890 cm^−1^, which are the characteristic peaks of the polysaccharide backbone. Broad peaks around 3000 to 3600 cm^−1^ can be assigned to OH stretching frequency of chitosan. The peaks around 1640 and 1567 cm^−1^ correspond to amide I and amide II, respectively [14]. A slight variation in stretching frequency was observed in the composite hydrogel. This might be because the pi-bonds of the carbon nanotube may be chemically interacting with the amide and OH groups of chitosan. However, most of the peaks belong to chitosan moieties.

In the present study, chitosan acts as a media to disperse the CNT solution, which can then act as antimicrobial agents. The OH stretching frequency of CNT-Chitosan composite hydrogels decreased from 3449 to 3434, 3432 and 3421 cm^−1^. This might be due to some kind of ionic bond formation, which may be possible between the pi bonds of the carbon nanotube with chitosan.

### 2.3. Scanning Electron Microscopy Observations of the Hydrogels

**Figure 3.**
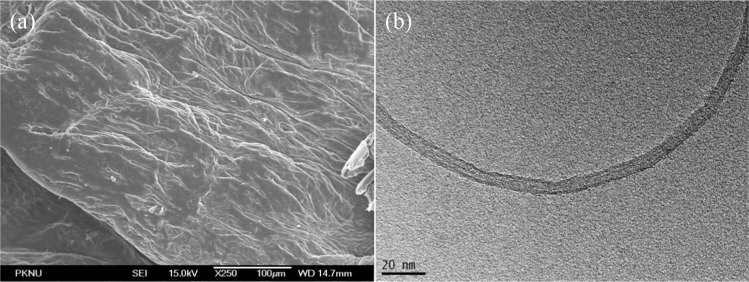
FE-SEM images of chitosan (**a**) bar is 100μm and TEM images of pristine carbon nanotube (**b**) bar is 20 nm.

Field emission scanning electron microscopy images of chitosan and pristine CNT are shown in Figure 3. Chitosan morphology was smooth and dense. The diameters of the carbon nanotubes are measured in nanometers.

**Figure 4.**
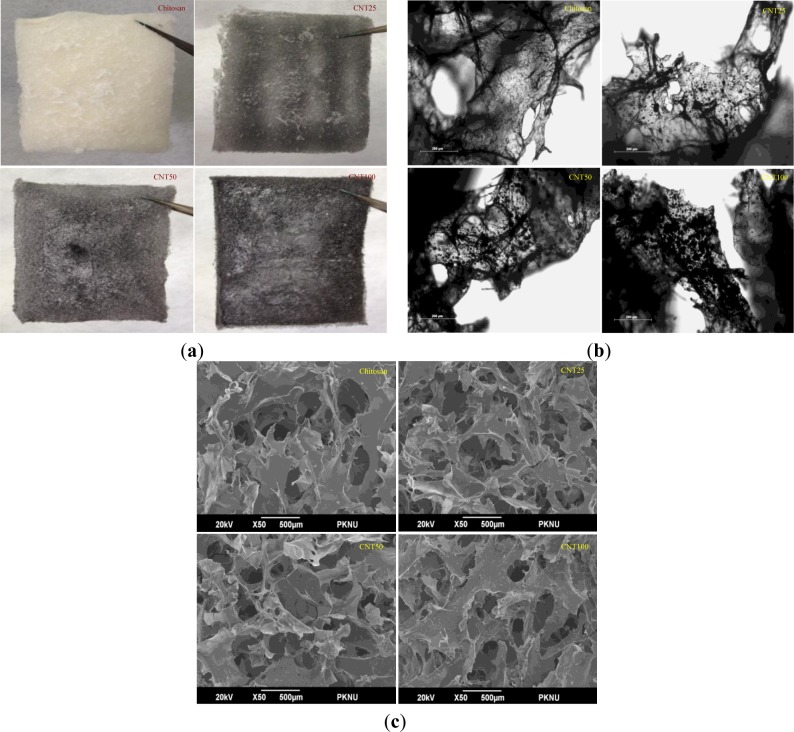
(**a**) Normal photograph images of CH, CNT25, CNT50 and CNT100. Sample size is 7 cm × 7cm, length and breadth; (**b**) Optical microscopic images of CH, CNT25, CNT50 and CNT100. Scale bar is 200 μm; (**c**) Scanning electron micrograph of CH, CNT25, CNT50 and CNT100. Scale bar is 500 μm.

For further confirmation, optical microscopy was performed to check the dispersion of CNT particles. A normal photograph of hydrogels is shown in Figure 4a. It was observed from the figures that chitosan hydrogel appeared to be white in color. On the other hand, CNT composites were black in color, and the color intensified even further with the increasing concentration of CNT.

Figure 4b clearly indicates that with an increase in the CNT concentration, deepened black color was observed, which further indicates that the CNT was uniformly dispersed in the chitosan matrix. Surface morphology, porosity, dispersion and microstructure of the hydrogels were examined by scanning electron microscopy, as presented in Figure 4c.

From the electron microscopic analysis, it was observed that all the hydrogels showed similar porosity level. The total porosity of the hydrogels were greater than 90% and water uptake and retention ability of the chitosan hydrogel was greater when compared to composite hydrogels. The hydrogels showed different surface appearance based on CNT concentration and the distribution and the size of their pores were also found to be different.

The distribution of the porosity was consistent with being condensed by increasing concentrations of CNT. From the results, it can be inferred that the fabricated hydrogel material is suitable for various biomedical and food science applications.

### 2.4. Antimicrobial Activity

**Figure 5.**
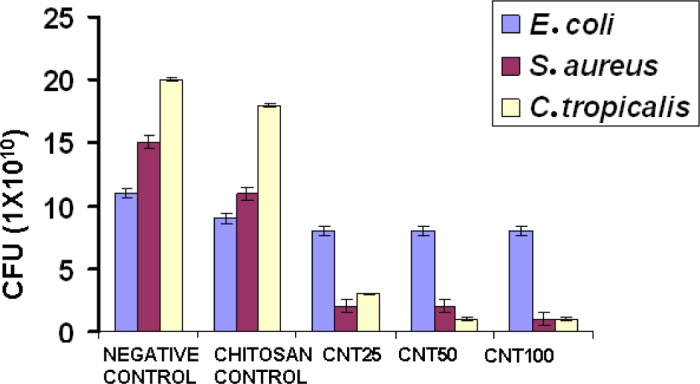
Antimicrobial activity of chitosan, CNT25, CNT50 and CNT100 against *E. coli* and *S. aureus* and *C. tropicalis*.

Figure 5 shows the antimicrobial activity of chitosan and its hydrogels. Compared with chitosan, all the CNT hydrogels showed higher antimicrobial activity. Numerous mechanisms have already been published pertaining to the antimicrobial activity of chitosan. One of the most accepted mechanisms is that the positive charge of chitosan probably interacts with negatively charged microbial cell membranes. The interaction occurs between protonated ammonium group of chitosan and the microbial cell surface, provoking the osmotic imbalance and at the same time CNT shows a synergistic effect that damages the membrane *in situ* to control the overall growth of the microbe. In another theory, it brings about the hydrolysis of intracellular peptidoglycans in the microbes [14]. The experiment shows that CNT incorporated hydrogel showed greater antimicrobial activity with respect to increasing concentration towards *S. aureus* when compared to *E. coli* and *C. tropicalis*. The three different concentrations of CNT composite hydrogels did not show considerable activity towards *E. coli*.

The presence of an outer membrane in *E. coli* might have caused obstruction in binding of CNT to initiate antimicrobial activity, which might be because of the strong net negative charge on the *E. coli* surface. The mechanism of effective antimicrobial activity towards *S. aureus* is through direct binding of bacterial surface proteins with the CNTs. The nanotubes get released from the hydrogel and target the bacteria, probably within 24 h, which further causes the microbial cell membrane lysis and subsequent loss of membrane potential [23,24]. Gram positive bacterium *S. aureus* was inhibited by higher molecular weight of chitosan. The main reason for this might be that the higher molecular weight of chitosan inhibits the nutrient adsorption. On the other hand, gram negative bacteria was not inhibited by higher molecular weight chitosan. This might be due to the fact that higher molecular weight chitosan cannot disturb the metabolism of the cell. The results are coherent with the previous literature [25].

## 3. Experimental Section

### 3.1. Materials

Chitosan with a degree of deacetylation of 90% and a molecular weight of 310 KDa was purchased from Kitto chemicals, Seoul, Korea. Multiwalled carbon nanotube (MWNT) outer diameter <8nm, length (10–30μm) were purchased from cheaptubes.com, Grafton, VT, USA. All the other chemicals and reagents were of analytical grade.

### 3.2. Hydrogels Preparation

Chitosan solution was prepared by dissolving it in 2% (V/V) acetic acid aqueous solvent. The solution was then filtered to remove undissolved particles. Chitosan hydrogel was prepared by raising the pH of chitosan solution to neutral by the addition of 1% (V/V) NaOH solution. Hydrogel was centrifuged at 2500RPM for 10 min, followed by the removal of supernatant.

For Chi-CNT hydrogel (CNT25), 25 mg of pristine multi walled carbon nanotubes were dissolved in 40 mL of distilled water. The CNT solution was dropped in chitosan gels drop wise to mix equally. The mixture was stirred at 850 rpm for 24 h at room temperature. After 24 h, hydrogel was kept to freeze at −24°C and dried. For CNT50 and CNT100; 50 and 100 mg of CNT were used, respectively.

### 3.3. Characterization

#### 3.3.1. Porosity Test

The porosity of hydrogels was measured by liquid displacement method as reported earlier [21] and water uptake and retention ability of the composite hydrogels were measured as per the previous report [26].

#### 3.3.2. General Characterization

The stretching frequencies of hydrogels were examined by Fourier transform infrared spectroscopy (FT-IR) (Perkin Elmer Inc., Waltham, MA, USA) and spectrum GX spectrometry within the range of 400 to 4000 cm^−1^. Morphology and pore size of chitosan and Chitosan-CNT hydrogels were obtained by optical microscopy (CTR 6000; Leica, Wetzlar, Germany) and scanning electron microscopy (SEM JSM-6490LV, JEOL, Peabody, MA, USA). The surface of hydrogels was sputtered with gold and photographed in the case of SEM analysis.

### 3.4. Antimicrobial Activity

Serial dilution method was performed to evaluate the antimicrobial activity towards *S. aureus*, *E. coli* and *C. tropicalis*. Microbial colony was inoculated overnight. The OD was compared to Mc Farland standard and equal volume of counted colonies was incubated for 24 h with carbon nanotubes (CNT) incorporated chitosan hydrogel of different concentrations 25, 50 and 100 mg and chitosan control. The samples were kept in a shaking incubator followed by serial dilution of each sample. The diluted samples were plated and the colonies formed were counted.

## 4. Conclusions

The developed Chitosan-CNT hydrogel exhibited well-defined pore structure with water uptake and retention ability. In addition, Chitosan-CNT hydrogel showed a strong antimicrobial activity against *S. aureus*, *E. coli* and *C. tropicalis*. Hence, we suggest that the CNT dispersed in a chitosan matrix will be a promising material for biomedical as well as food science applications.
